# Supplementary material for: Effects of Antioxidant Vitamins, Curry Consumption, and Heavy Metal Levels on Metabolic Syndrome with Comorbidities: A Korean Community-Based Cross-Sectional Study
Source: Antioxidants (Basel). 2021 May 19;10(5):808. doi: 10.3390/antiox10050808 (PMC8161368; doi:10.3390/antiox10050808)
Supplement: Supplementary file 1 [file antioxidants-10-00808-s001.zip › antioxidants-1190761-supplementary.pdf]

Effects of antioxidant vitamins, curry consumption and heavy metal levels on the metabolic syndrome with comorbidities: a Korean community-based cross-sectional study

Hai Nguyen Duc, MD [1], Hojin Oh, PharmD [1], Min-Sun Kim, PhD [1]

[1] Department of Pharmacy, College of Pharmacy and Research Institute of Life and Pharmaceutical Sciences, Sunchon National University, Sunchon, Jeonnam, Republic of Korea

Corresponding author:

Prof. Min-Sun Kim PhD**,**

Department of Pharmacy, College of Pharmacy, Sunchon National University, Sunchon 57922, Republic of Korea**,**

Email: minsun@scnu.ac.kr.

Tel: +821025101635

Supplementary material

Table S1. Adjusted ORs for serum lead (Pb).

Metabolic Syndrome	Odds ratio	95% Confident Interval		*p*-value
**Serum Pb** *(μg/L)*	1.144	1.026	1.276	0.015
**Monthly household income** *(<2,000)*	1 (refer)	.	.	.
≥ 2,000 and < 4,000	0.976	0.734	1.299	0.869
≥ 4,000 and < 6,000	0.854	0.619	1.179	0.337
≥ 6,000	0.727	0.513	1.030	0.073
**Residential areas** *(urban)*	1	.	.	.
Rural	1.171	0.890	1.541	0.258
**Energy** *(Kcal)*	1.000	1.000	1.000	0.099
**Age group** *(29 years)*	1	.	.	.
30-39	2.233	1.328	3.753	0.002
40-49	5.697	3.494	9.289	<0.001
50-59	6.504	3.897	10.854	<0.001
>60	7.738	4.389	13.643	<0.001
**Occupation** *(Managers, professional)*	1	.	.	.
Office worker, clerical workers	0.965	0.619	1.504	0.876
Service workers, sales workers	0.974	0.649	1.462	0.900
Agriculture, forestry and fishing workers	0.761	0.424	1.366	0.361
Craft, plant and machine operators and assemblers	0.585	0.364	0.940	0.027
Elementary occupations	0.791	.49	1.275	0.336
Unemployed	0.906	.626	1.311	0.599
**Sex** *(male)*	1	.	.	.
Female	3.980	2.897	5.467	<0.001
**Family history of CVDs** *(no)*	1	.	.	.
Yes	1.251	1.008	1.552	0.042
**Family history of type 2 diabetes** *(no)*	1	.	.	.
Yes	1.146	0.979	1.342	0.090
**Family history of hyperlipidemia** *(no)*	1	.	.	.
Yes	1.130	0.755	1.690	0.553

**BMI group** *(<18.5)*	1	.	.	.
≥ 18·5 and < 25	4.488	1.227	16.418	0.023
≥ 25 and < 30	22.784	6.206	83.65	<0.001
≥ 30	56.738	14.795	217.585	<0.001
**Smoking status** *(non/ex-smoker)*	1	.	.	.
Current smoker	1.194	0.875	1.630	0.264
**High risk drinking** *(no)*	1	.	.	.
Yes	0.951	0.689	1.313	0.760
**Physical activity** *(Not regular)*	1	.	.	.
Regular	0.857	0.666	1.103	0.232
**Education level** (*≤ Middle school*)	1	.	.	.
High school	0.857	0.666	1.103	0.232
>= College	0.857	0.666	1.103	0.232
**Hypertension** *(no)*	1	.	.	.
yes	3.646	2.724	4.88	<0.001
**Dyslipidemia** *(no)*	1	.	.	.
Yes	2.288	1.695	3.088	<0.001
**Diabetes** *(no)*	1	.	.	.
Yes	3.131	2.031	4.826	<0.001
**Stroke** *(no)*	1	.	.	.
Yes	2.091	0.822	5.318	0.122
**MI or angina** *(no)*	1	.	.	.
Yes	2.588	0.067	100.67	0.611
**MI** *(no)¶*	1	.	.	.
Yes	0.398	0.017	9.467	0.569
**Angina** *(no)*	1	.	.	.
Yes	0.478	0.037	6.205	0.572
**Arthritis** *(no)*	1	.	.	.
Yes	0.478	0.037	6.205	0.572
**Osteoarthritis** *(no)*	1	.	.	.
yes	1.290	0.102	16.314	0.844
**Rheumatoid arthritis** *(no)*	1	.	.	.
Yes	2.476	0.211	29.049	0.471
**Kidney failure** *(no)*	1	.	.	.
Yes	1.378	0.247	7.697	0.715
**Depression** *(no)*	1	.	.	.
Yes	1.507	0.951	2.388	0.081

**Thyroid disease** *(no)*	1	.	.	.
Yes	0.936	0.530	1.655	0.821
**Asthma** *(no)*	1	.	.	.
Yes	0.923	0.473	1.804	0.816
**Green vegetable consumption** *(low)*	1	.	.	.
High	1.053	0.829	1.337	0.674
**White vegetable consumption** *(low)*	1	.	.	.
High	1.009	0.777	1.310	0.947
**Fruit consumption** *(low)*	1	.	.	.
High	0.905	0.721	1.137	0.390

**Mean dependent var**	0.169	**SD dependent var**	0.375
**Pseudo r-squared**	0.327	**Number of obs**	3948
**Chi-square**	1176.431	**Prob > chi2**	0.000
**Akaike crit. (AIC)**	2508.388	**Bayesian crit. (BIC)**	2797.313

¶**MI:** myocardial infarction

Table S2. Adjusted ORs for serum mercury (Hg).

Metabolic Syndrome	Odds ratio	95% Confident Interval		*p*-value
**Serum Hg** *(μg/L)*	1.033	1.021	1.064	0.031
**Monthly household income** *(<2,000)*	1 (Ref)	.	.	.
≥ 2,000 and < 4,000	0.949	0.714	1.262	0.718
≥ 4,000 and < 6,000	0.827	0.600	1.141	0.248
≥ 6,000	0.698	0.493	0.988	0.043
**Residential areas** *(urban)*	1	.	.	.
Rural	1.182	0.899	1.555	0.232
**Energy** *(Kcal)*	1	1	1	0.944
**Age group** *(29 years)*	1	.	.	.
30-39	2.253	1.34	3.786	0.002
40-49	5.839	3.586	9.507	<0.001
50-59	6.798	4.090	11.297	<0.001
>60	8.042	4.576	14.133	<0.001
**Occupation** *(Managers, professional)*	1	.	.	.
Office worker, clerical workers	0.967	0.620	1.506	0.880
Service workers, sales workers	0.996	0.663	1.495	0.984
Agriculture, forestry and fishing workers	0.756	0.421	1.358	0.349
Craft, plant and machine operators and assemblers	0.616	0.384	0.989	0.045
Elementary occupations	0.798	0.494	1.288	0.355
Unemployed	0.919	0.634	1.332	0.656
**Sex** *(male)*	1	.	.	.
Female	3.911	2.851	5.364	<0.001
**Family history of CVDs** *(no)*	1	.	.	.
Yes	1.274	1.027	1.581	0.028
**Family history of type 2 diabetes** *(no)*	1	.	.	.
Yes	1.007	0.792	1.281	0.954
**Family history of hyperlipidemia** *(no)*	1	.	.	.
Yes	1.143	0.764	1.711	0.515
**BMI group** *(<18.5)*	1	.	.	.
≥ 18·5 and < 25	4.056	1.163	14.148	0.028
≥ 25 and < 30	20.48	5.846	71.74	<0.001

≥ 30	48.913	13.367	178.99	<0.001
**Smoking status** *(non/ex-smoker)*	1	.	.	.
Current smoker	1.237	0.907	1.687	0.178
**High risk drinking** *(no)*	1	.	.	.
Yes	0.965	0.699	1.331	0.826
**Physical activity** *(Not regular)*	1	.	.	.
Regular	0.859	0.667	1.105	0.235
**Education level** (*≤ Middle school*)	1	.	.	.
High school	0.972	0.726	1.302	0.851
>= College	0.513	0.358	0.735	<0.001
**Hypertension** *(no)*	1	.	.	.
yes	3.64	2.719	4.872	<0.001
**Dyslipidemia** *(no)*	1	.	.	.
Yes	2.262	1.676	3.053	<0.001
**Diabetes** *(no)*	1	.	.	.
Yes	3.079	2.001	4.737	<0.001
**Stroke** *(no)*	1	.	.	.
Yes	2.165	0.844	5.554	0.108
**MI or angina** *(no)*	1	.	.	.
Yes	2.785	0.072	107.683	0.583
**MI** *(no)¶*	1	.	.	.
Yes	0.433	0.019	10.067	0.602
**Angina** *(no)*	1	.	.	.
Yes	0.478	0.015	14.867	0.674
**Arthritis** *(no)*	1	.	.	.
Yes	0.556	0.046	6.746	0.645
**Osteoarthritis** *(no)*	1	.	.	.
yes	1.110	0.094	13.122	0.934
**Rheumatoid arthritis** *(no)*	1	.	.	.
Yes	2.146	0.196	23.506	0.532
**Kidney failure** *(no)*	1	.	.	.
Yes	1.326	0.236	7.468	0.749
**Depression** *(no)*	1	.	.	.
Yes	1.480	0.935	2.342	0.094
**Thyroid disease** *(no)*	1	.	.	.
Yes	0.919	0.521	1.622	0.771
**Asthma** *(no)*	1	.	.	.
Yes	0.930	0.478	1.808	0.830

**Green vegetable consumption** *(low)*	1	.	.	.
High	1.055	0.831	1.340	0.661
**White vegetable consumption** *(low)*	1	.	.	.
High	0.995	0.766	1.292	0.970
**Fruit consumption** *(low)*	1	.	.	.
High	0.902	0.718	1.133	0.375

**Mean dependent var**	0.169	**SD dependent var**	0.375
**Pseudo r-squared**	0.327	**Number of obs**	3948
**Chi-square**	1175.198	**Prob > chi2**	0.000
**Akaike crit. (AIC)**	2509.621	**Bayesian crit. (BIC)**	2798.545

¶**MI:** myocardial infarction

Table S3. Adjusted ORs for serum c-reactive protein (CRP).

**Metabolic Syndrome**	**Odds ratio**	**95% Confident Interval**		***p*-value**
**Serum CRP** *(mg/L)*	1.089	1.036	1.144	0.001
**Monthly household income** *(<2,000)*	1	.	.	.
≥ 2,000 and < 4,000	1.041	0.718	1.509	0.833
≥ 4,000 and < 6,000	0.904	0.611	1.338	0.615
≥ 6,000	0.804	0.538	1.202	0.288
**Residential areas** *(urban)*	1	.	.	.
Rural	1.019	0.744	1.395	0.906
**Energy** *(Kcal)*	1	1	1	0.579
**Age group** *(29 years)*	1	.	.	.
30-39	2.36	1.286	4.334	0.006
40-49	4.927	2.739	8.862	<0.001
50-59	5.794	3.165	10.608	<0.001
>60	5.451	2.771	10.723	<0.001
**Occupation** *(Managers, professional)*	1	.	.	.
Office worker, clerical workers	0.979	0.612	1.566	0.930
Service workers, sales workers	0.878	0.552	1.395	0.582
Agriculture, forestry and fishing workers	0.962	0.443	2.088	0.922
Craft, plant and machine operators and assemblers	0.964	0.575	1.614	0.889
Elementary occupations	0.897	0.512	1.572	0.704
Unemployed	1.024	0.687	1.527	0.906
**Sex** *(male)*	1	.	.	.
Female	3.84	2.676	5.511	<0.001
**Family history of CVDs** *(no)*	1	.	.	.
Yes	1.287	1.003	1.652	0.048
**Family history of type 2 diabetes** *(no)*	1	.	.	.
Yes	1.249	.968	1.610	0.087
**Family history of hyperlipidemia** *(no)*	1	.	.	.
Yes	.933	.637	1.368	0.724
**BMI group** *(<18.5)*	1	.	.	.
≥ 18·5 and < 25	4.447	0.996	19.86	0.051
≥ 25 and < 30	23.179	5.176	103.808	<0.001
≥ 30	50.156	10.791	233.125	<0.001
**Smoking status** *(non/ex-smoker)*	1	.	.	.

Current smoker	1.662	1.170	2.306	0.005
**High risk drinking** *(no)*	1	.	.	.
Yes	0.761	0.525	1.103	0.149
**Physical activity** *(Not regular)*	1	.	.	.
Regular	0.997	0.755	1.315	0.981
**Education level** (≤ *Middle school*)	1	.	.	.
High school	1.116	0.778	1.601	0.549
>= *College*	.683	0.450	1.037	0.073
**Hypertension** *(no)*	1	.	.	.
yes	4.477	3.248	6.170	<0.001
**Dyslipidemia** *(no)*	1	.	.	.
Yes	2.307	1.67	3.187	<0.001
**Diabetes** *(no)*	1	.	.	.
Yes	3.909	2.372	6.441	<0.001
**Stroke** *(no)*	1	.	.	.
Yes	1.711	0.569	5.147	0.339
**MI or angina** *(no)*	1	.	.	.
Yes	4.891	0.049	486.909	0.499
**MI** *(no)¶*	1	.	.	.
Yes	0.036	0.001	1.333	0.071
**Angina** *(no)*	1	.	.	.
Yes	0.301	0.004	23.762	0.590
**Arthritis** *(no)*	1	.	.	.
Yes	0.071	0.005	1.045	0.054
**Osteoarthritis** *(no)*	1	.	.	.
yes	7.810	0.549	111.192	0.129
**Rheumatoid arthritis** *(no)*	1	.	.	.
Yes	13.615	1.116	166.046	0.041
**Kidney failure** *(no)*	1	.	.	.
Yes	0.022	0.001	0.349	0.007
**Depression** *(no)*	1	.	.	.
Yes	1.447	0.843	2.484	0.180
**Thyroid disease** *(no)*	1	.	.	.
Yes	1.704	1.005	2.887	0.048
**Asthma** *(no)*	1	.	.	.
Yes	1.536	0.723	3.262	0.264
**Green vegetable consumption** *(low)*	1	.	.	.
High	1.117	0.851	1.466	0.424

**White vegetable consumption** *(low)*	1	.	.	.
High	0.855	0.632	1.158	0.311
**Fruit consumption** *(low)*	1	.	.	.
High	0.844	0.647	1.102	0.213

**Mean dependent var**	0.188	**SD dependent var**	0.391
**Pseudo r-squared**	0.328	**Number of obs**	2888
**Chi-square**	914.365	**Prob > chi2**	0.000
**Akaike crit. (AIC)**	1966.468	**Bayesian crit. (BIC)**	2241.011

Table S4. Adjusted ORs for vitamin B1 intake.

Metabolic Syndrome	Odds ratio	95% Confident Interval		*p*-value
**Vitamin B1 intake** *(mg)*	0.884	0.792	0.974	0.013
**Monthly household income** *(<2,000)*	1	.	.	.
≥ 2,000 and < 4,000	0.900	0.745	1.086	0.271
≥ 4,000 and < 6,000	.847	0.687	1.043	0.118
≥ 6,000	.732	0.583	0.918	0.007
**Residential areas** *(urban)*	1	.	.	.
Rural	1.121	.939	1.339	0.207
**Energy** *(Kcal)*	1	1	1	0.071
**Age group** *(29 years)*	1	.	.	.
30-39	2.430	1.685	3.503	<0.001
40-49	5.029	3.544	7.138	<0.001
50-59	6.687	4.666	9.585	<0.001
>60	7.173	4.835	10.641	<0.001
**Occupation** *(Managers, professional)*	1	.	.	.
Office worker, clerical workers	1.106	0.829	1.476	0.494
Service workers, sales workers	1.051	0.804	1.374	0.715
Agriculture, forestry and fishing workers	0.905	0.613	1.334	0.613
Craft, plant and machine operators and assemblers	0.896	0.659	1.219	0.486
Elementary occupations	0.776	0.564	1.069	0.121
Unemployed	0.979	0.769	1.246	0.861
**Sex** *(male)*	1	.	.	.
Female	3.939	3.202	4.846	<0.001
**Family history of CVDs** *(no)*	1	.	.	.
Yes	1.089	.947	1.252	0.232
**Family history of type 2 diabetes** *(no)*	1	.	.	.
Yes	1.169	1.002	1.364	0.048
**Family history of hyperlipidemia** *(no)*	1	.	.	.
Yes	1.059	0.818	1.371	0.665
**BMI group** *(<18.5)*	1	.	.	.
≥ 18·5 and < 25	8.748	2.743	27.896	<0.001
≥ 25 and < 30	43.398	13.587	138.614	<0.001

≥ 30	113.66	34.857	370.624	<0.001
**Smoking status** *(non/ex-smoker)*	1	.	.	.
Current smoker	1.282	1.045	1.575	0.017
**High risk drinking** *(no)*	1	.	.	.
Yes	0.976	0.792	1.204	0.822
**Physical activity** *(Not regular)*	1	.	.	.
Regular	0.905	0.771	1.062	0.221
**Education level** (*≤ Middle school*)	1	.	.	.
High school	0.939	0.775	1.138	0.523
>= College	0.615	0.486	0.779	<0.001
**Hypertension** *(no)*	1	.	.	.
yes	3.98	3.321	4.770	<0.001
**Dyslipidemia** *(no)*	1	.	.	.
Yes	2.388	1.968	2.898	<0.001
**Diabetes** *(no)*	1	.	.	
Yes	3.53	2.677	4.653	<0.001
**Stroke** *(no)*	1	.	.	.
Yes	1.019	0.587	1.767	0.947
**MI or angina** *(no)*	1	.	.	.
Yes	0.260	0.020	3.333	0.301
**MI** *(no)¶*	1	.	.	.
Yes	1.693	0.189	15.146	0.638
**Angina** *(no)*	1	.	.	.
Yes	4.328	0.371	50.546	0.243
**Arthritis** *(no)*	1	.	.	.
Yes	0.119	0.029	.489	0.003
**Osteoarthritis** *(no)*	1	.	.	.
yes	6.240	1.548	25.151	0.010
**Rheumatoid arthritis** *(no)*	1	.	.	.
Yes	6.403	1.732	23.677	0.005
**Kidney failure** *(no)*	1	.	.	.
Yes	0.838	0.353	1.991	0.689
**Depression** *(no)*	1	.	.	.
Yes	1.499	1.111	2.022	0.008
**Thyroid disease** *(no)*	1	.	.	.
Yes	1.376	1.009	1.878	0.044
**Asthma** *(no)*	1	.	.	.
Yes	1.173	0.770	1.788	0.456

**Green vegetable consumption** *(low)*		1	.	.	.
High		1.043	0.894	1.217	0.591
**White vegetable consumption** *(low)*		1	.	.	.
High		0.904	0.764	1.070	0.240
**Fruit consumption** *(low)*		1	.	.	.
High		0.873	0.754	1.010	0.068
**Mean dependent var**	0.180	**SD dependent var**			0.384
**Pseudo r-squared**	0.324	**Number of obs**			8944
**Chi-square**	2734.303	**Prob > chi2**			0.000
**Akaike crit. (AIC)**	5793.258	**Bayesian crit. (BIC)**			6119.800

Table S5. Adjusted ORs for vitamin C intake.

Metabolic Syndrome	Odds ratio	95% Confident Interval		*p*-value
**Vitamin C intake** *(mg)*	0.991	0.982	0.994	0.032
**Monthly household income** *(<2,000)*	1	.	.	.
≥ 2,000 and < 4,000	0.906	0.751	1.094	0.306
≥ 4,000 and < 6,000	.855	.694	1.053	0.140
≥ 6,000	.743	.592	.932	0.010
**Residential areas** *(urban)*	1	.	.	.
Rural	1.115	0.934	1.331	0.228
**Energy** *(Kcal)*	1	1	1	0.420
**Age group** *(29 years)*	1	.	.	.
30-39	2.443	1.694	3.521	<0.001
40-49	5.084	3.582	7.217	<0.001
50-59	6.839	4.768	9.811	<0.001
>60	7.382	4.972	10.96	<0.001
**Occupation** *(Managers, professional)*	1	.	.	.
Office worker, clerical workers	1.113	0.834	1.486	0.466
Service workers, sales workers	1.048	0.802	1.371	0.730
Agriculture, forestry and fishing workers	0.919	0.623	1.355	0.669
Craft, plant and machine operators and assemblers	0.891	0.655	1.213	0.464
Elementary occupations	.771	0.560	1.062	0.111
Unemployed	0.98	0.770	1.248	0.871
**Sex** *(male)*	1	.	.	.
Female	4.017	3.263	4.945	<0.001
**Family history of CVDs** *(no)*	1	.	.	.
Yes	1.089	0.947	1.252	0.232
**Family history of type 2 diabetes** *(no)*	1	.	.	.
Yes	1.165	0.998	1.359	0.053
**Family history of hyperlipidemia** *(no)*	1	.	.	.
Yes	1.068	0.825	1.382	0.619
**BMI group** *(<18.5)*	1	.	.	.
≥ 18·5 and < 25	8.813	2.763	28.117	<0.001

≥ 25 and < 30	43.807	13.709	139.985	<0.001
≥ 30	113.145	34.685	369.083	<0.001
**Smoking status** *(non/ex-smoker)*	1	.	.	.
Current smoker	1.279	1.042	1.571	0.019
**High risk drinking** *(no)*	1	.	.	.
Yes	0.966	0.784	1.191	0.748
**Physical activity** *(Not regular)*	1	.	.	.
Regular	0.910	0.775	1.068	0.248
**Education level** (*≤ Middle school*)	1	.	.	.
High school	0.945	0.780	1.146	0.565
>= College	0.619	0.488	0.784	<0.001
**Hypertension** *(no)*	1	.	.	.
yes	3.97	3.313	4.758	<0.001
**Dyslipidemia** *(no)*	1	.	.	.
Yes	2.393	1.972	2.904	<0.001
**Diabetes** *(no)*	1	.	.	
Yes	3.524	2.674	4.645	<0.001
**Stroke** *(no)*	1	.	.	.
Yes	1.019	0.587	1.767	0.948
**MI or angina** *(no)*	1	.	.	.
Yes	0.264	0.020	3.411	0.308
**MI** *(no)¶*	1	.	.	.
Yes	1.677	0.187	15.069	0.645
**Angina** *(no)*	1	.	.	.
Yes	4.347	0.369	51.17	0.243
**Arthritis** *(no)*	1	.	.	.
Yes	0.127	0.031	.518	0.004
**Osteoarthritis** *(no)*	1	.	.	.
yes	5.865	1.463	23.519	0.013
**Rheumatoid arthritis** *(no)*	1	.	.	.
Yes	6.096	1.655	22.458	0.007
**Kidney failure** *(no)*	1	.	.	.
Yes	0.841	0.355	1.995	0.695
**Depression** *(no)*	1	.	.	.
Yes	1.505	1.116	2.031	0.007
**Thyroid disease** *(no)*	1	.	.	.
Yes	1.395	1.022	1.903	0.036
**Asthma** *(no)*	1	.	.	.

Yes	1.167	0.766	1.778	0.472
**Green vegetable consumption** *(low)*	1	.	.	.
High	1.043	0.893	1.217	0.596
**White vegetable consumption** *(low)*	1	.	.	.
High	0.908	0.768	1.075	0.263
**Fruit consumption** *(low)*	1	.	.	.
High	0.872	0.753	1.009	0.066
**Mean dependent var**	0.180	**SD dependent var**	0.384	
**Pseudo r-squared**	0.324	**Number of obs**	8944	
**Chi-square**	2732.715	**Prob > chi2**	0.000	
**Akaike crit. (AIC)**	5794.846	**Bayesian crit. (BIC)**	6121.388	

Table S6. Adjusted ORs for curry consumption.

Metabolic Syndrome	Odds ratio	95% Confident Interval		*p*-value
**Curry consumption** *(low)*	1 (refer)	.	.	.
High	0.853	0.740	0.983	0.028
**Monthly household income** *(<2,000)*	1	.	.	.
≥ 2,000 and < 4,000	0.907	0.751	1.095	0.309
≥ 4,000 and < 6,000	0.851	0.691	1.049	0.130
≥ 6,000	0.733	0.585	0.920	0.007
**Residential areas** *(urban)*	1	.	.	.
Rural	1.112	0.932	1.328	0.240
**Energy** *(Kcal)*	1.000	1.000	1.000	0.842
**Age group** *(29 years)*	1	.	.	.
30-39	2.426	1.684	3.495	<0.001
40-49	4.944	3.489	7.005	<0.001
50-59	6.440	4.500	9.216	<0.001
>60	6.924	4.673	10.261	<0.001
**Occupation** *(Managers, professional)*	1	.	.	.
Office worker, clerical workers	1.107	0.830	1.477	0.489
Service workers, sales workers	1.047	0.801	1.369	0.737
Agriculture, forestry and fishing workers	0.904	0.613	1.334	0.611
Craft, plant and machine operators and assemblers	0.894	0.657	1.215	0.474
Elementary occupations	0.774	0.562	1.065	0.116
Unemployed	0.974	0.765	1.239	0.828
**Sex** *(male)*	1	.	.	.
Female	3.916	3.186	4.813	<0.001
**Family history of CVDs** *(no)*	1	.	.	.
Yes	1.086	0.945	1.249	0.246
**Family history of type 2 diabetes** *(no)*	1	.	.	.
Yes	1.179	1.011	1.376	0.036
**Family history of hyperlipidemia** *(no)*	1	.	.	.
Yes	1.068	0.825	1.382	0.619
**BMI group** *(<18.5)*	1	.	.	.
≥ 18·5 and < 25	8.682	2.720	27.711	<0.001
≥ 25 and < 30	43.12	13.488	137.85	<0.001
≥ 30	112.002	34.32	365.517	<0.001

**Smoking status** *(non/ex-smoker)*	1	.	.	.
Current smoker	1.308	1.066	1.605	0.010
**High risk drinking** *(no)*	1	.	.	.
Yes	0.984	0.798	1.213	0.879
**Physical activity** *(Not regular)*	1	.	.	.
Regular	0.899	0.766	1.055	0.192
**Education level** (≤ *Middle school*)	1	.	.	.
High school	0.930	0.768	1.127	0.460
>= *College*	0.619	0.489	0.784	<0.001
**Hypertension** *(no)*	1	.	.	.
yes	3.989	3.329	4.780	<0.001
**Dyslipidemia** *(no)*	1	.	.	.
Yes	2.382	1.963	2.89	<0.001
**Diabetes** *(no)*	1	.	.	.
Yes	3.563	2.706	4.691	<0.001
**Stroke** *(no)*	1	.	.	.
Yes	1.014	0.586	1.755	0.960
**MI or angina** *(no)*	1	.	.	.
Yes	0.231	0.018	2.902	0.256
**MI** *(no)¶*	1	.	.	.
Yes	1.905	0.217	16.691	0.561
**Angina** *(no)*	1	.	.	.
Yes	4.751	0.415	54.429	0.210
**Arthritis** *(no)*	1	.	.	.
Yes	0.124	0.030	0.502	0.003
**Osteoarthritis** *(no)*	1	.	.	.
yes	6.016	1.509	23.988	0.011
**Rheumatoid arthritis** *(no)*	1	.	.	.
Yes	6.16	1.682	22.557	0.006
**Kidney failure** *(no)*	1	.	.	.
Yes	0.848	0.359	2.007	0.708
**Depression** *(no)*	1	.	.	.
Yes	1.497	1.109	2.021	0.008
**Thyroid disease** *(no)*	1	.	.	.
Yes	1.404	1.029	1.914	0.032
**Asthma** *(no)*	1	.	.	.
Yes	1.169	0.767	1.782	0.469
**Mean dependent var**	0.180	**SD dependent var**		0.384

**Pseudo r-squared**	0.323	**Number of obs**	8944
**Chi-square**	2726.634	**Prob > chi2**	0.000
**Akaike crit. (AIC)**	5794.927	**Bayesian crit. (BIC)**	6100.173
